# A murine model of diarrhea, growth impairment and metabolic disturbances with *Shigella flexneri* infection and the role of zinc deficiency

**DOI:** 10.1080/19490976.2018.1564430

**Published:** 2019-02-03

**Authors:** Pedro Henrique Q.S. Medeiros, Solanka E. Ledwaba, David T. Bolick, Natasa Giallourou, Lauren K. Yum, Deiziane V.S. Costa, Reinaldo B. Oriá, Eileen M. Barry, Jonathan R. Swann, Aldo Ângelo M. Lima, Hervé Agaisse, Richard L. Guerrant

**Affiliations:** a Center for Global Health, Division of Infectious Diseases and International Health, University of Virginia, Charlottesville, USA; b Institute of Biomedicine, Federal University of Ceara, Fortaleza, Brazil; c Division of Computational and Systems Medicine, Department of Surgery and Cancer, Imperial College London, London, UK; d Department of Microbiology, Immunology and Cancer Biology, University of Virginia, Charlottesville, USA; e Center for Vaccine Development, University of Maryland, Baltimore, USA

**Keywords:** Shigellosis, mouse model, zinc deficiency, urine metabolomics, intestinal microbiota

## Abstract

*Shigella* is one of the major enteric pathogens worldwide. We present a murine model of *S. flexneri* infection and investigate the role of zinc deficiency (ZD). C57BL/6 mice fed either standard chow (HC) or ZD diets were pretreated with an antibiotic cocktail and received *S. flexneri* strain 2457T orally. Antibiotic pre-treated ZD mice showed higher *S. flexneri* colonization than non-treated mice. ZD mice showed persistent colonization for at least 50 days post-infection (pi). *S. flexneri*-infected mice showed significant weight loss, diarrhea and increased levels of fecal MPO and LCN in both HC and ZD fed mice. *S. flexneri* preferentially colonized the colon, caused epithelial disruption and inflammatory cell infiltrate, and promoted cytokine production which correlated with weight loss and histopathological changes. Infection with *S. flexneri ΔmxiG* (critical for type 3 secretion system) did not cause weight loss or diarrhea, and had decreased stool shedding duration and tissue burden. Several biochemical changes related to energy, inflammation and gut-microbial metabolism were observed. Zinc supplementation increased weight gains and reduced intestinal inflammation and stool shedding in ZD infected mice. In conclusion, young antibiotic-treated mice provide a new model of oral *S. flexneri* infection, with ZD promoting prolonged infection outcomes.

## Introduction

*Shigella* is an important, inflammatory enteric pathogen responsible for significant burden of diarrhea worldwide.^1^ Recent multicenter epidemiologic studies have highlighted the association of *Shigella* with both moderate to severe and community diarrhea in children under 24 months old.^2,3^ The development of molecular diagnostic techniques has revealed a substantially greater prevalence of *Shigella* infections, which were not seen in the past using much less sensitive culturing methods.^4,5^ Moreover, the lack of a vaccine, despite multiple and diverse vaccine design strategies, and antimicrobial resistance are major challenges for controlling shigellosis.^6^

Information is rapidly emerging on *Shigella* virulence factors associated with disease and its elicited host responses, especially through studies using guinea pig, rabbit ileal loop and murine pulmonary models.^7–9^ However, these approaches are limited for not assessing colonic tissue and/or not allowing for oral infection.^10^ Other models have also been proposed but their drawbacks include costs (as in the macaque monkey model) and complexity (as in subcutaneous human colon xenografts).^10^ In addition, many efforts have been made for developing a robust mouse model that could allow oral administration of the inoculum, but only few studies have been published and have not yet fully characterized host responses and common clinical outcomes.^11,12^

A clearer understanding of the virulence mechanisms among the different *Shigella* species is warranted to design better interventions, such as vaccines.^13,14^ Moreover, the poor understanding of the influence of environmental factors on the virulence of *Shigella* infection is well documented.^1,10^ Data addressing the influence of a host-undernourished state on microbial virulence, host inflammatory response and vaccine efficacy are missing. Indeed, diverse kinds of undernutrition are highly prevalent in settings where shigellosis has a substantial burden.^2^

In this study, in order to further address these questions, we developed a mouse model of infection with oral inoculation of *S. flexneri* serotype 2a strain (the most prevalent serotype in developing settings) and evaluated the impact of zinc deficiency on this infection. We have characterized clinical outcomes – bodyweight growth and diarrhea, bacterial colonization, biochemical perturbations and inflammatory immune responses.

## Results

### Antibiotic treatment and zinc deficiency increased *S. flexneri* stool shedding in mice

**Figure 1.**
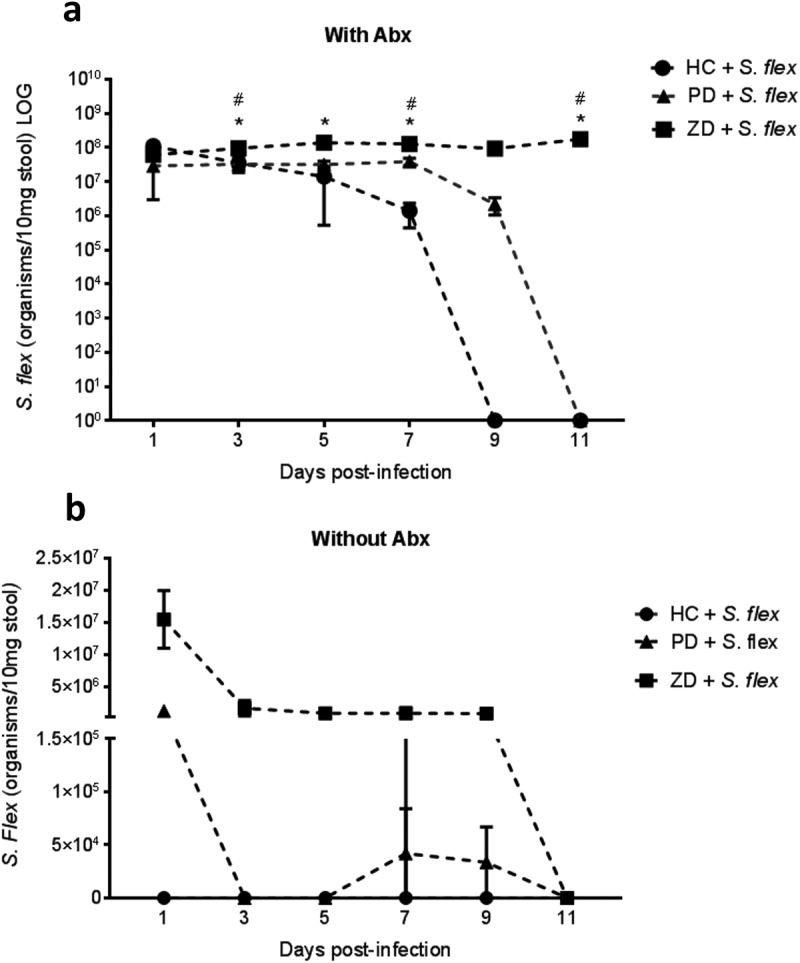
Antibiotic treatment and zinc deficiency increased *S. flexneri* stool shedding.C57BL/6 mice were fed house chow (HC), protein deficient (PD) and zinc-deficient (ZD) defined diets for 2 weeks and submitted or not to antibiotic cocktail in the drinking water for 3 d before oral infection with 10^8^ CFU/mouse *S. flexneri* 2457T strain. DNA extracted from stools after infection were analyzed by *ipaH* gene qPCR. (**a)** Stool shedding using antibiotics (with Abx) across HC, PD and ZD diets. * P < 0.05 by two-way ANOVA, HC + *S. flex* vs ZD + *S. flex*, # P < 0.05 by two-way ANOVA, PD + *S. flex* vs ZD + *S flex* (N = 4/group); (**b)** Stool shedding without using antibiotics (without Abx) across HC, PD and ZD diets (N = 4/group). Antibiotic pre-treated nourished C57BL/6 mice were evaluated for bodyweight growth change and signs of diarrhea induced by oral *S. flexneri* 2457T infection (N = 8/group). Data were replicated at least two times. N = 8/group.

To induce susceptibility to infection, we tested the use of a broad antibiotic cocktail consisting of metronidazole, colistin, gentamicin and vancomycin given in the drinking water for 3 d before removal one day prior to infection. Concomitantly, we tested the use of three different diets for 2 weeks prior infection that were then maintained throughout the experiment: house chow (control “nourished”), protein and zinc-deficient defined diets. Overall, antibiotic pre-treated mice showed higher *S. flexneri* stool shedding than non-antibiotic treated mice. Antibiotic treated house chow-fed mice began to stop shedding after day 7 pi, followed by protein-deficient mice after day 9 pi. Zinc-deficient mice continued to shed persistently at robust levels – about 10^8^ organisms/10 mg stool until at least 50 d pi. Stool shedding levels of zinc-deficient mice were significantly increased on days 3, 5, 7 and 11 pi when compared to house chow-fed mice (P < 0.05 by two-way ANOVA). Stool shedding in zinc-deficient mice was also significantly greater than that in protein-deficient mice at days 3, 7 and 11 pi (P < 0.05 by two-way ANOVA) (Figure 1(a)). Non-antibiotic treated protein-deficient mice had low stool shedding for 1 week, while non-antibiotic treated house chow-fed mice did not shed at all throughout the experiment. Zinc-deficient mice without antibiotics had robust stool shedding levels for 9 d, but lower than with antibiotic treatment (about 10^6^ organisms/10 mg stool) (Figure 1(b)). Model development continued with a focus on antibiotic pre-treated house chow-fed mice in comparison with zinc-deficient mice.

### *S. flexneri* orally administered in antibiotic pre-treated house chow-fed and zinc-deficient mice caused acute growth impairment, diarrhea and intestinal inflammation

**Figure 2.**
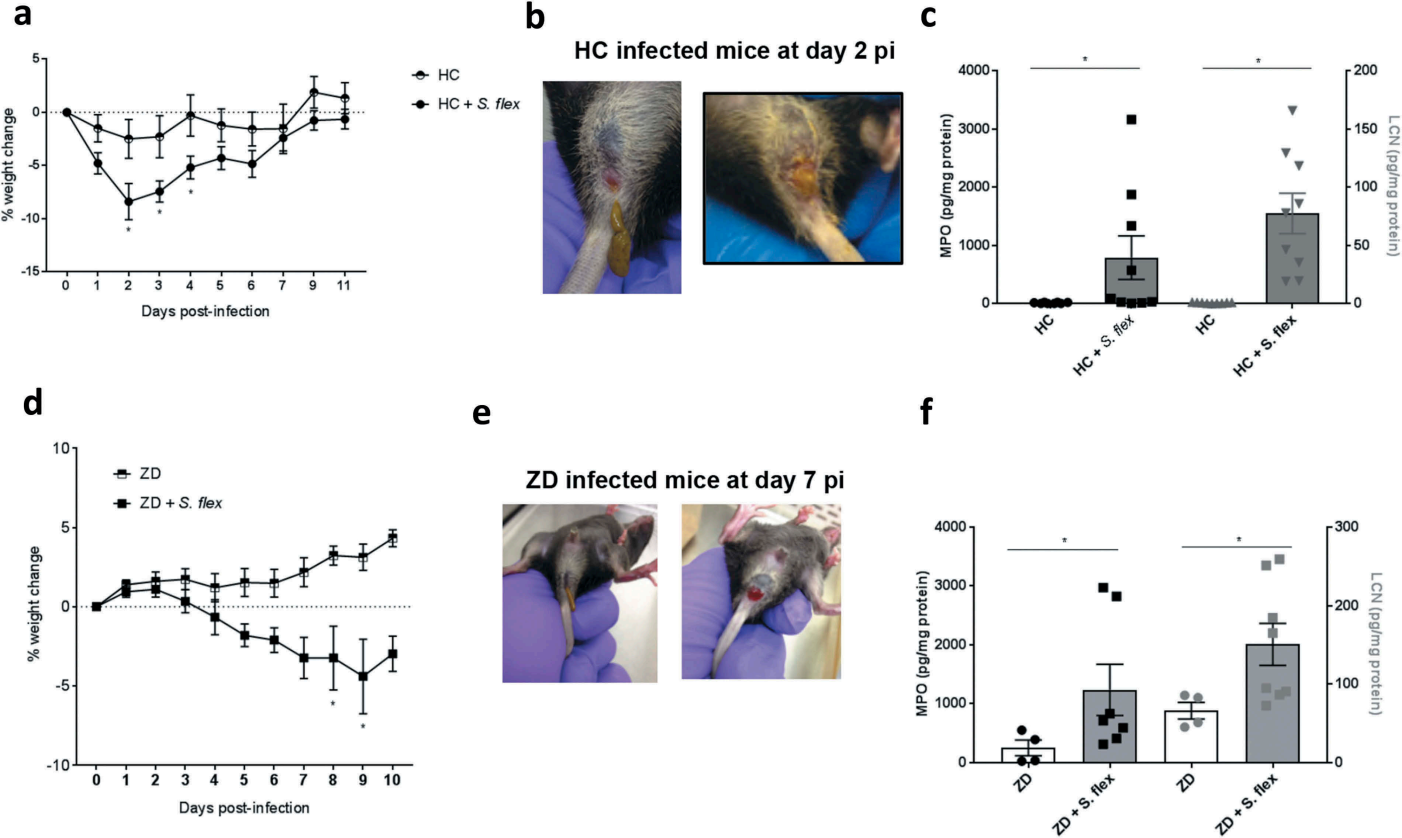
***S. flexneri* orally administered in antibiotic pre-treated house chow-fed and zinc-deficient mice caused acute growth impairment, diarrhea and intestinal inflammation**. Mice previously exposed to 2 weeks on house chow or zinc-deficient diet and pre-treated with antibiotic cocktail in the drinking water were challenged with *S. flexneri* 10^8^ CFU/mouse inoculum. Bodyweight growth change and signs of diarrhea were observed, and specimens were collected for protein extraction and measurement of biomarkers of intestinal inflammation – myeloperoxidase (MPO) and lipocalin (LCN) by ELISA kits (**a)**. Growth impairment induced by *S. flexneri* on days 2–4 in nourished mice. * P < 0.05 by two-way ANOVA, HC vs HC + S. flex. (**b)** Representative pictures of diarrhea in nourished mice at day 2 pi. (**c)** Cecal contents MPO and LCN levels from day 7 pi in house chow-fed mice. * P = 0.005 for MPO and P = 0.0006 for LCN by Mann–Whitney test, HC vs HC + S. flex. (**d)** Growth impairment induced by *S. flexneri* on days 8–9 pi in zinc-deficient mice. * P < 0.05 by two-way ANOVA, ZD vs ZD + *S. flex*. (**e)** Representative pictures of diarrhea in nourished mice at day 7 pi. (**f)** Cecal contents MPO and LCN levels from day 7 pi in zinc-deficient mice. * P = 0.042 for MPO and P = 0.016 for LCN by Mann–Whitney test, ZD vs ZD + S. flex. Data were replicated at least two times. N = 8/group.

After observing *S. flexneri* colonization in antibiotic pre-treated mice, we then evaluated disease outcomes associated with *S. flexneri* infection – following bodyweight and signs of diarrhea. In antibiotic pre-treated house chow-fed mice, oral 10^8^ CFU/mouse *S. flexneri* infection caused acute weight loss and diarrhea within 1–4 d pi. Significant bodyweight differences between control and infected groups were observed (P < 0.05 by two-way ANOVA at days 2, 3 and 4 pi) (Figure 2(a)). Across different experiments, the decrements in the bodyweight could vary within 5–15% of bodyweight at the moment of infection. Figure 2(b) shows representative pictures of diarrhea in antibiotic-treated house chow-fed mice. Diarrhea was not seen in antibiotic non-pretreated mice. Mice generally recovered after day 5 pi from both growth impairment and from diarrhea. When mice that were fed a zinc-deficient diet for 2 weeks prior to infection were challenged with same *S. flexneri* 10^8^ CFU/mouse inoculum, they also showed weight loss and diarrhea but over a different time frame compared with house chow-fed mice. Infected zinc-deficient mice had significant weight loss when compared to zinc-deficient controls (P < 0.01 by two-way ANOVA at days 8 and 9 pi) (Figure 2(d)). Figure 2(e) shows representative pictures of diarrhea observed in infected zinc-deficient mice. Despite a more prolonged duration of disease in zinc-deficient infected mice, most mice recovered after 1 week of disease. The timing of the peak of disease outcomes varied among mice independent of which diet they received. Regarding intestinal inflammatory biomarkers, myeloperoxidase (MPO) and lipocalin-2 (LCN) levels from cecal contents were significantly increased in house chow-fed and mice at day 7 pi (MPO: P = 0.0051 and LCN: P = 0.0006: by Mann–Whitney test) (Figure 2(c)). Similarly, infected zinc-deficient mice also showed increased MPO and LCN (MPO: P = 0.042 and LCN: P = 0.016 by Mann–Whitney test) (Figure 2(f)).

### *S. flexneri* 2457T preferentially colonized the colon and caused intestinal epithelial damage

**Figure 3.**
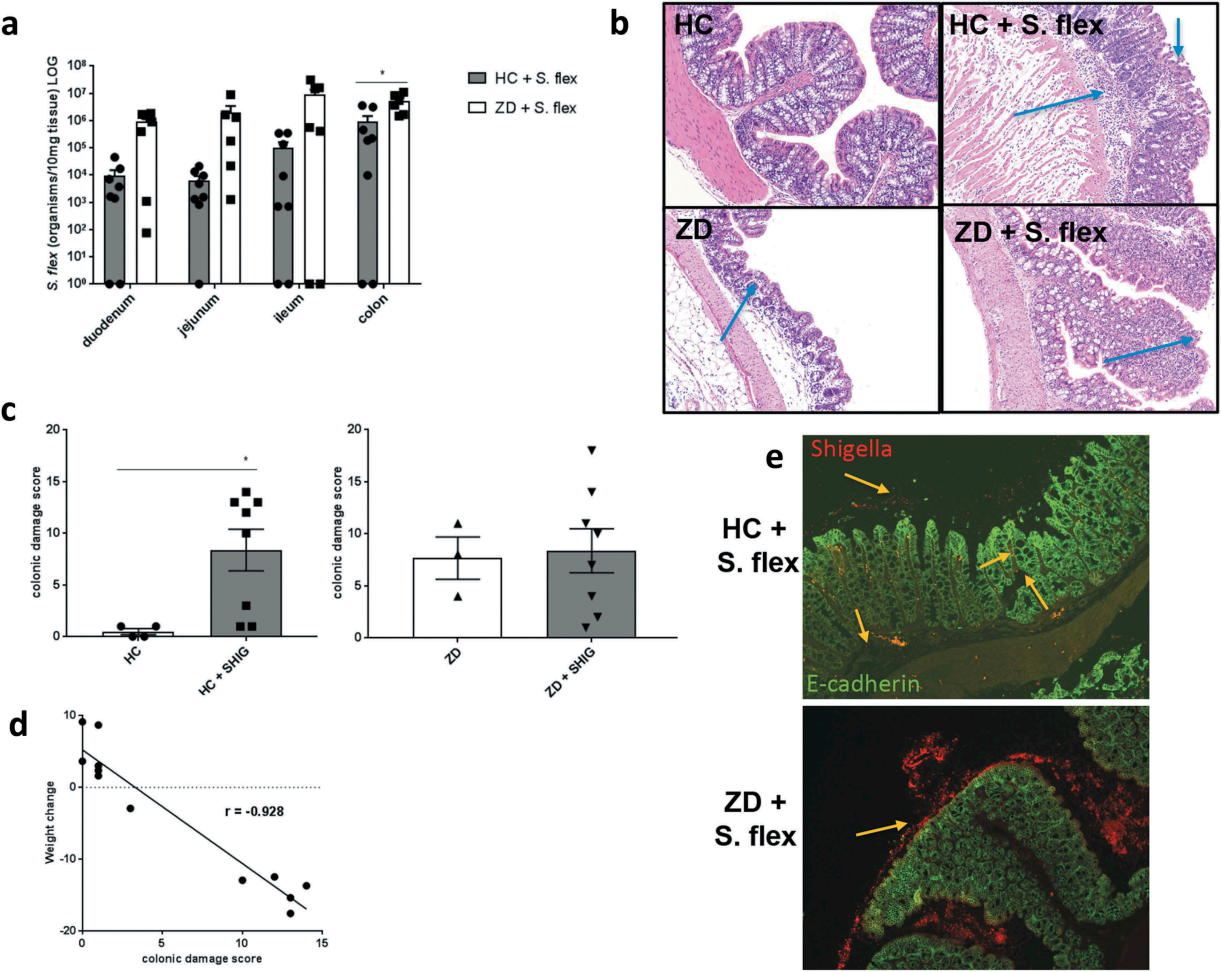
***S. flexneri* 2457T preferentially colonized the colon and caused intestinal epithelial damage**. *S. flexneri* infected nourished and zinc-deficient C57BL/6 mice were euthanized and intestinal specimens collected. *S. flexneri* target molecular quantification of DNA extracted from different intestinal sections from both nourished and zinc-deficient infected mice at day 3 pi. Colon sections from infected zinc-deficient mice were stained for H&E, as well as for *S. flexneri* specific and E-cadherin (epithelium marker) antibodies. (**a)** *S. flexneri* tissue burden levels across duodenum, jejunum, ileum and colon at day 3 pi. * P = 0.008 by Mann–Whitney test, HC + *S. flex* vs ZD + *S. flex* in the colon. **(b)** Representative picture of H&E staining of colon section from infected and uninfected house chow-fed and zinc-deficient mice. Arrows indicate epithelial damage and inflammatory cell infiltrate. **(c)** Analysis of histopathological scoring of colon from infected and uninfected house chow-fed mice. * P = 0.018 by Mann–Whitney test. **(d)** Analysis of histopathological scoring of colon from infected and uninfected zinc-deficient mice. (**e)** Representative picture of stained colon sections showing *S. flexneri* in red and E-cadherin in green, indicating by arrows *S. flexneri* present adhered to mucosa and epithelium, as well as in the lamina propria in house chow-fed mice (on top); and abundant extracellular *S. flexneri* in zinc-deficient mice (on bottom). Data were replicated at least two times. N = 4/group.

In order to evaluate the bowel region *Shigella* were colonizing mice, we performed *S. flexneri* targeted molecular quantification of DNA extracted from different intestinal sections from both house chow-fed mice and zinc deficient infected mice at day 3 pi. Zinc deficient mice showed increased levels of *S. flexneri* when compared to house chow-fed mice in the colon (P = 0.008 by Mann–Whitney test). For both groups, colon was the most colonized section (Figure 3(a)). Further, we analyzed H&E histopathology of the colon at peaks of infection in mice fed either house-chow or zinc-deficient diets. Figure 3(b) shows colon pictures of uninfected mice and infected mice, with clear epithelial damage, inflammatory cell infiltrate and vascular hemorrhage. After analysis for histopathology scoring, there was a significant difference between infected and uninfected house-chow-fed mice (P = 0.018 by Mann–Whitney test), with no alteration by infection in zinc-deficient mice (Figure 3(c)). Further, colonic damage score was inversely correlated with weight change among nourished mice (P < 0.0001, r = −0.928 by Spearman test) (Figure 3(d)). *S. flexneri* was found to be predominant in the lumen of the colon with some rare bacteria observed in the lamina propria in house chow-fed mice, while it was highly predominant as biofilm-like structures in close contact with epithelial cells in zinc-deficient mice (Figure 3(e)) (**Figure S1** shows a higher magnified picture of the *S. flexneri* stained colon section from a zinc deficient mouse). Of note, infected zinc deficient mice showed detectable *S. flexneri* levels in systemic tissues at day 3 pi. Specifically, 2/8 blood samples, 6/8 spleen samples and 8/8 liver samples from zinc-deficient mice were positive for *S. flexneri* by qPCR. On the other hand, no infected house chow-fed mice samples showed *S. flexneri* in the same locations.

### Challenge with the *S. flexneri* Δmxig mutant strain did not lead to weight loss, pathogen colonization or intestinal inflammation

**Figure 4.**
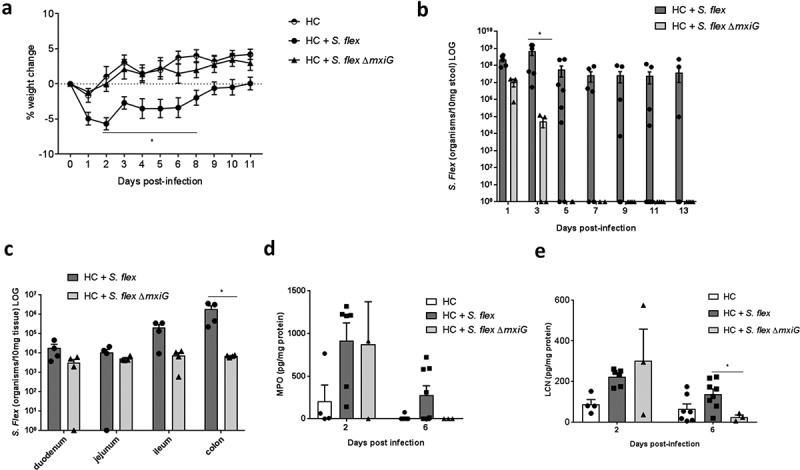
**Challenge with the** *S. flexneri ΔmxiG* **did not lead to weight loss, pathogen colonization and intestinal inflammation**. Mice were infected by either a wild type or *ΔmixG S. flexneri* 2457T strain. Bodyweights changes, diarrhea, stool shedding, tissue burden and intestinal inflammation were analyzed. (**a)** Bodyweight growth change induced by *S. flexneri ΔmixG* in antibiotic pre-treated nourished C57BL/6 mice. * P < 0.05 by two-way ANOVA, HC + *S. flex* vs HC + *S. flex ΔmixG*. **(b)** *S. flexneri* stool shedding comparing wild type *S. flexneri* and *S. flexneri ΔmixG* until day 9 pi. * P = 0.0095 by Mann-Whitney test, HC + *S. flex* vs HC + *S. flex ΔmixG*. at day 3pi. (**c)** *S. flexneri* tissue burden of duodenum, jejunum, ileum and colon comparing wild-type *S. flexneri* and *S. flexneri ΔmixG* at day 3 pi. * P = 0.0286 by Mann–Whitney test. (**d)** Fecal MPO and LCN levels of mice infected by wild-type *S. flexneri* and *S. flexneri ΔmixG* at days 2 and 6 pi. * P < 0.05 by one-way ANOVA. Data were replicated at least two times. N = 4/group.

We next investigated whether the infection outcomes were caused by specific *S. flexneri* virulence properties. By performing infection with the *S. flexneri ΔmxiG* 2457T strain in parallel the wild-type strain infection, antibiotic pre-treated house chow-fed mice receiving the mutant strain did not suffer the same consequences as mice infected by the wild-type strain. Bodyweight following *S. flexneri ΔmxiG* mutant strain infection was not different from the non-infected controls and was significantly different from the mice infected with wild type *S. flexneri* (P < 0.05 by two-way ANOVA) (Figure 4(a)). Stool shedding of the *S. flexneri ΔmxiG* strain lasted only until day 3 pi, while stool shedding of the wild-type strain lasted for more than 10 d pi. Specifically, at day 3 pi, stool shedding levels of the *S. flexneri ΔmxiG* strain were significantly decreased (P = 0.0095 by Mann–Whitney test) (Figure 4(b)). In addition, day 3 pi tissue burden was consistently slightly lower with the *S. flexneri ΔmxiG* strain across different sections of the intestine, with colonic *S. flexneri ΔmxiG* strain abundance being highly significantly lower compared to wild type *S. flexneri* abundance (P = 0.0286 by Mann–Whitney test) (Figure 4(c)). Intestinal inflammation induced by the *S. flexneri ΔmxiG* strain, as measured by MPO and LCN fecal levels, was not different from the wild type infected group on day 2 pi but was significantly lower on day 6 pi (P < 0.05 by 1way ANOVA) (Figure 4(d)).

### *S. flexneri* triggered cytokine immune responses in both house chow and zinc-deficient mice

**Figure 5.**
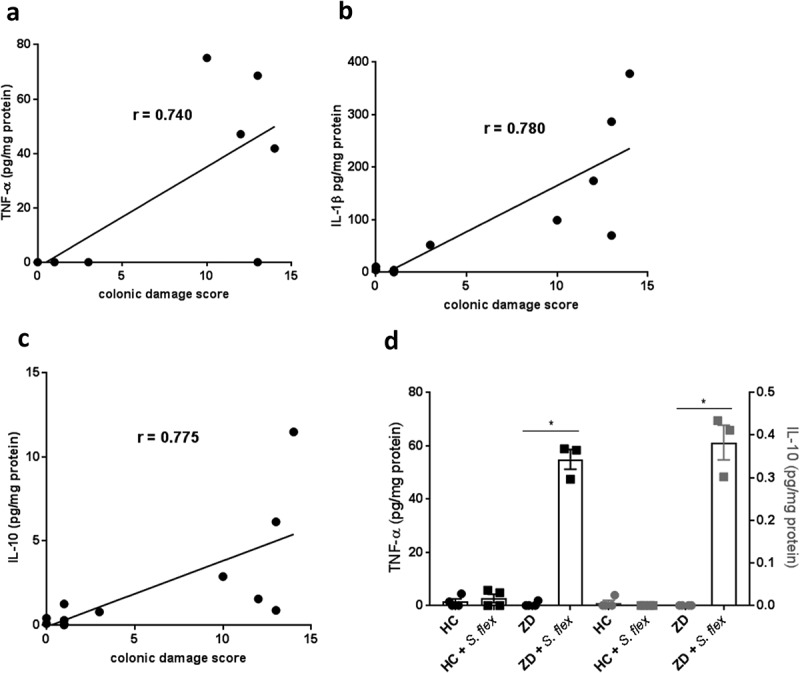
***S. flexneri* triggered cytokine immune responses in both house chow and zinc-deficient mice**. Total protein was extracted from intestinal sections for immune response markers analysis at day 15 pi. **(a)** Correlation between colonic TNF-α protein levels and colonic damage score at day 3 pi in house chow-fed mice, r = 0.640, P = 0.0285. (**b)** Correlation between colonic IL-1β protein levels and colonic damage score at day 3 pi in house chow-fed mice, r = 0.780, P = 0.004. (**c)** Correlation between colonic IL-10 protein levels and colonic damage score at day 3 pi in house chow-fed mice, r = 0.775, P = 0.004. (**d)** TNF-α and IL-10 protein levels from the cecal contents at day 15 pi in house chow-fed and zinc-deficient mice after *S. flexneri* infection. * P = 0.043 for TNF-α ZD vs ZD + *S. flex* and * P = 0.286 for IL-10 ZD vs ZD + *S. flex* by Mann–Whitney test. Data were replicated at least two times. N = 4–8/group.

For evaluating immune responses to *S. flexneri* in the model, we extracted protein from intestinal sections for immune response markers analysis. While cytokine levels varied among individual mice, TNF-α, IL-1β and IL-10 protein levels from colon were significantly correlated with colonic damage score at day 3 pi in house chow-fed mice (P = 0.0285, r = 0.640 for TNF-α; P = 0.004, r = 0.780 for IL-1β; and P = 0.004, r = 0.775 for IL-10 by Spearman tests), but not in zinc-deficient mice (Figure 5(a-c)). These immune markers were also inversely correlated with weight change at day 3 pi (P = 0.0431, r = −0.599 for TNF-α; P = 0.015, r = −0.693 for IL-1β; and P = 0.0142, r = −0.6993 for IL-10 by Spearman tests) and other inflammatory markers detected in the colon (macrophage inflammatory protein 2 – MIP-2, monocyte chemoattractant protein 1- MCP1, KC – IL-8 homolog and granulocyte-colony stimulating factor – G-CSF) also showed significant correlations with histopathological scores and weight changes (data not shown). Further, TNF-α protein levels from the cecal contents at day 15 pi were also significantly increased with *S. flexneri* infection but only in the zinc deficient mice (P = 0.043 by Mann–Whitney test). Similarly, IL-10 protein levels from cecal contents were also higher in zinc-deficient infected mice (P = 0.0286 by Mann–Whitney test) (Figure 5(d)). At the same time point, TNF-α, IFN-γ, IL-4 and TLR-4 mRNA levels from ileum and colon showed similar trends, but were not significantly different (data not shown).

### Zinc supplementation reduced *S. flexneri* stool shedding, improved bodyweight and decreased intestinal inflammation in zinc-deficient mice

**Figure 6.**
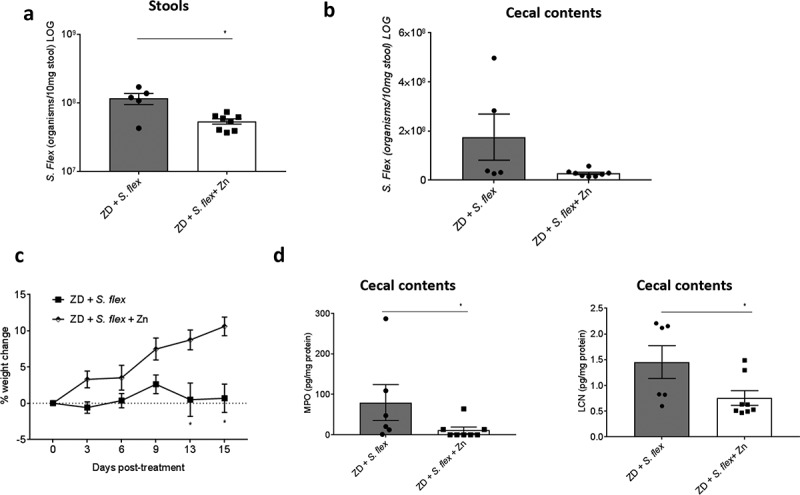
**Zinc supplementation reduced *S. flexneri* stool shedding and intestinal inflammation, and improved bodyweight growth in zinc-deficient mice**. *S. flexneri* infected zinc-deficient mice started to receive zinc treatment at day 33 pi and followed until day 48 pi (total of 15 d). Zinc sulfate was dissolved in water and given in the drinking water. Stool shedding, bodyweight growth and intestinal inflammation myeloperoxidase (MPO) and lipocalin-2 (LCN) cecal contents levels were analyzed. (**a)** *S. flexneri* stool shedding of infected zinc-deficient mice receiving zinc or not after 15 d. * P = 0.0295 by Mann–Whitney test. (**b)** *S. flexneri* burden in the cecal contents of infected zinc-deficient mice receiving zinc or not after 15 d. (**c)** Bodyweight growth of infected zinc-deficient mice receiving zinc or not after 15 d. * P < 0.0001 by two-way ANOVA at day 15 post-treatment. (**d)** MPO and LCN levels from cecal contents at day 15 post-treatment. * P < 0.05 by Mann–Whitney test, ZD + *S. flex* vs ZD + *S. flex* + Zn. Data were replicated at least two times. N = 8/group.

Due to the persistent colonization of *S. flexneri* in zinc deficient mice, we decided to test whether zinc supplementation could reduce pathogen burden and improve *S. flexneri*-related effects in zinc deficient infected mice. At day 33 pi, zinc was given in the drinking water and mice were analyzed 15 d of treatment (day 48 pi). Mice receiving zinc showed significantly less stool shedding than non-treated zinc-deficient mice (P = 0.0295 by Mann–Whitney test) (Figure 6(a)). *S. flexneri* number of organisms from the cecal contents showed the same trend, albeit not significant (P = 0.0653 by Mann–Whitney test) (Figure 6(b)). Zinc supplementation also improved bodyweight by 10% of infected zinc-deficient mice (P < 0.0001 by two-way ANOVA on day 15 post-treatment) (Figure 6(c)). Intestinal inflammation was also assessed and mice receiving zinc showed significantly lower levels of MPO and LCN (Figure 6(d)) than non-treated mice at day 15 post-treatment (P < 0.05 by Mann–Whitney test).

### Metabolic perturbations induced by *S. flexneri* infection and malnutrition

**Figure 7.**
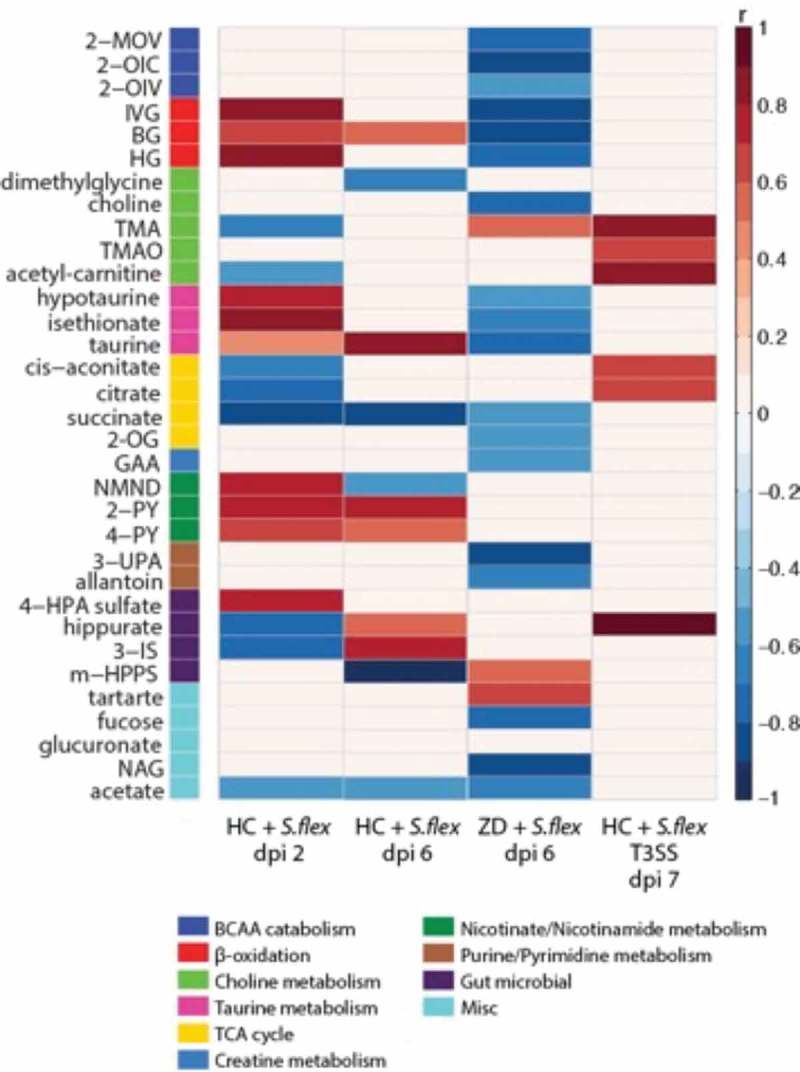
**Metabolic perturbations induced by *S. flexneri* infection**. Heat map summarizing the significant urinary metabolic alterations induced by *S.flexneri* identified by OPLS-DA models. Results are presented as correlation coefficients (r). Red color indicates increased excretion and blue indicated decreased excretion of urinary metabolites in nourished mice infected at day 2 and day 6 versus diet and age-matched uninfected mice, in zinc-deficient infected mice at day 6 compared to diet and age-matched controls and in nourished mice infected with the *S. flexneri ΔmixG* strain versus mice infected with the wild type strain. Abbreviations: 2-OG, 2-oxoglutarate, 2-OIV, 2-oxoisovalerate, 2-OIC, 2-oxoisocaproate, 2-MOV, 3-methyl-2-oxovalerate, 2-PY, *N*-methyl-2-pyridone-5-carboxamide, 3-IS, 3indoxylsulfate, 3-UPA, 3-ureidopropionic acid, 4-HPA, 4-hydroxyphenylacetate, 4-PY, *N*-methyl-4-pyridone-3-carboxamide, BG, butyrylglycine, GAA guanidinoacetate, HG, hexanoylglycine, IVG, isovalerylglycine, m-HPPS, m-hydroxyphenylpropiobylsulfate, NAG, N-acetyl glutamine, NMND, *N*-methylnicotinamide, TMA, trimethylamine, TMAO, trimethylamine-N-oxide. Data were replicated at least two times. N = 8/group.

In order to further investigate potential biochemical pathways related to *S. flexneri* pathobiology in this model, we assessed the urinary metabolic perturbations induced by *S. flexneri* infection in house chow-fed and zinc-deficient mice. The metabolic signature of infection with the *S. flexneri ΔmxiG* was also compared with *S. flexneri* 2457T infection. Consistent with the disease outcomes with *S. flexneri* infection, significant alterations to the urinary metabolic profiles were observed 2 d after infection in the house chow-fed mice compared to uninfected mice (Q^2^Ŷ = 0.37, *p* = 0.03) but not in the infected zinc-deficient mice. However, after 6 d of infection, significant alterations in the urinary metabolic profiles did emerge in the zinc-deficient mice (Q^2^Ŷ = 0.65, *p* = 0.02) while biochemical disturbances remained in the infected house chow-fed mice (Figure 7).

Greater excretion of tryptophan-kynurenine derivatives, *N*-methylnicotinamide (NMND), *N*-methyl-2-pyridone-5-carboxamide (2-PY) and *N*-methyl-4-pyridone-3-carboxamide (4-PY) was observed in the infected house chow-fed mice 2 d pi indicating changes in energy expenditure and an inflammatory response of the host. Following 6 d pi, 2-PY and 4-PY remained elevated while NMND was excreted in lower amounts compared to the uninfected controls. Consistent with the biomarkers of intestinal inflammation, zinc deficient infected mice did not show altered excretion of these metabolites. Following 2 d of infection, house chow-fed mice excreted higher amounts of glycine conjugated intermediates of fatty acid β-oxidation (isovalerylglycine, butyrylglycine, hexanoylglycine) and lower amounts of acetylcarnitine, a metabolite related to the shuttling of fatty acids across the mitochondria. In the zinc-deficient mice, isovalerylglycine, butyrylglycine and hexanoylglycine were excreted in lower amounts 6 d after infection compared to uninfected zinc-deficient mice. Furthermore, 2 d pi house chow-fed animals excreted greater amounts of taurine and taurine-related metabolites (hypotaurine, isethionate) while these same metabolites were excreted in lower amounts 6 d pi in the zinc-deficient mice. As we have previously shown, zinc deficiency alone results in a lower excretion of BCAA catabolites (2-MOV, 2-OIC, 2-OIV).^15^ Following 6 d of *S. flexneri* infection, these BCAA catabolites were further reduced in the zinc deficient mice. Finally, urinary acetate excretion was lower following infection in both the zinc deficient and house chow-fed animals, likely due to fluctuations in the dynamics of the gut microbiome also demonstrated through shifts in the excretion of 4-HPA sulfate, 3-indoxyl sulfate and hippurate.

Metabolic disruptions were observed 7 d pi in the house chow-fed mice infected with the *S. flexneri ΔmxiG* strain compared to their uninfected controls (Q^2^Ŷ = 0.48, P = 0.03). The mutant resulted in an increased excretion of the microbial choline metabolites, trimethylamine (TMA) and trimethylamine*-N*-oxide, and the related metabolite, acetyl-carnitine. This was the opposite to the changes seen in the *S. flexneri 2457T* infected house chow-fed mice, which excreted lower amounts of TMA and acetyl-carnitine. Similarly, infection with the mutant strain increased the excretion of the TCA cycle intermediates, *cis*-aconitate and citrate and the microbial-host co-metabolite, hippurate 7 d pi. These metabolites were excreted in lower amounts in the house chow-fed animals following infection with the *S. flexneri 2457T* strain 2 d pi.

## Discussion

Shigellosis has been increasingly recognized worldwide and the need for better tools that improve testing of vaccines and other interventions is clearly evident.^14,16^ This is the first study that describes a murine model of intestinal shigellosis mimicking the clinical outcomes that are commonly seen in children, including diarrhea and weight loss, with host zinc deficiency promoting more prolonged infection.

Several efforts have been made to model shigellosis *in vivo*.^8,12,17–20^ However, unnatural inoculum administration routes and the absence of common disease outcomes are limitations of current models. In contrast, by using prior antibiotic treatment, we have been able to reproduce common human clinical effects through oral administration of *S. flexneri* inoculum, that are also affected by dietary zinc deficiency. Different antibiotic treatments lead to different susceptible conditions for the host;^21^ and association of selected bacterial species in germ-free mice leads to different interactions with *Shigella*.^22^ We have used a broad spectrum antibiotic cocktail to induce shigellosis susceptibility, in contrast to a study by Martino et al. who used only streptomycin.^19^ The use of antibiotics was crucial for enabling *S. flexneri* colonization in our mouse model. Indeed, *S. flexneri* inoculation in non-antibiotic treated house chow-fed mice did not provide robust colonization or outcomes. Other studies that used the same antibiotic cocktail have shown increased susceptibility to other experimental enteric infections.^23–26^

Similar to humans, *S. flexneri* preferentially colonized the colonic region of the intestine. In addition, accentuated intestinal inflammation and epithelial damage was observed in our experimental model and corroborates the pathobiology described for shigellosis in humans.^27^ *S. flexneri* presented as a highly inflammatory pathogen, being present either in the lamina propria or adherent to mucosa and epithelium in house chow-fed mice. Interestingly, *S. flexneri* was present predominantly in the extracellular area of the epithelium in zinc deficient mice. This observation could be related to the increased mucus production in zinc-deficient mice,^23^ that may block *S. flexneri* penetration into the intestinal epithelium, and suggests extracellular *S. flexneri* may also play a role in pathogenesis in mice. Moreover, emerging evidence of intestinal inflammation and growth impairment due to asymptomatic *Shigella* spp. has been changing classical perspectives in shigellosis.^28^

Another interesting finding of this study was the T3SS dependent pathology observed. In comparison with infection by *S. flexneri* wild type strain, abrogation of weight loss and diarrhea, less tissue burden, shorter stool shedding duration and decreased intestinal inflammation were observed when mice were infected with the *S. flexneri ΔmxiG* strain (critical for T3SS assembly). *S. flexneri* uses its T3SS apparatus for injecting virulence factors into the host cell, being a crucial step for the pathogenesis process.^29,30^ Results from our metabolomics experiments further substantiate the attenuated virulence of the *S. flexneri ΔmxiG* strain where metabolic perturbations induced by the wild-type strain (reduced excretion of choline-related metabolites, TCA cycle metabolites and hippurate) were reversed by the mutant. Parasitic infections have been previously associated with the reduced excretion of hippurate,^31^ a microbial-host co-metabolite resulting from the glycine conjugation of benzoic acid in the mitochondrial matrix. Reduced hippurate excretion is consistent with functional disturbances in the gut microbiome. In addition, consistent with our observations urinary hippurate excretion often covaries with the excretion of TCA cycle intermediates.^32^ It can therefore be inferred that mitochondrial functions remain intact in the *S. flexneri ΔmxiG* strain infected mice allowing for both TCA and hippurate formation to take place. Other *in vivo* models have shown the essential role of T3SS to *S. flexneri* virulence.^33,34^

In this model, while both house chow-fed and zinc deficient mice showed increased intestinal inflammation after infection with *S. flexneri*; an early elevated excretion of kynurenine/nicotinamide metabolites (2-PY and 4-PY) was observed only in house chow-fed, but not in zinc-deficient mice. Kynurenine metabolites are commonly described as important mediators of local and systemic immune response suppression.^35^ In addition, a previous study from our group showed zinc-deficient mice had fewer myeloid leukocytes than controls.^23^ Leukocytes are known to contain high amounts of taurine, which can have a role in acute or chronic immune responses as an antioxidant and cytoprotectant.^36^ Therefore, reduced leukocyte infiltration in zinc-deficient infected mice may reflect lower systemic availability of taurine and could help explain the reduced excretion of taurine related metabolites when compared with the house chow-fed infected mice. These metabolomic findings may help to explain the chronic effects of infection seen in zinc-deficient mice.

The development of normal immune response for faster clearance and recovery in shigellosis has been demonstrated in humans.^37^ In addition, this model also reflects key components of human shigellosis, such as increases on pro and anti-inflammatory cytokines profiles.^38,39^ Early cytokine production (TNF-α, IL-1β and IL-10) was seen in infected house chow-fed mice, and was correlated with weight change and histopathological scores, reinforcing their role in the pathogenesis and biomarkers of infection outcomes. In addition, the persistent *S. flexneri* colonization observed in zinc deficient mice was accompanied by TNF-α and IL-10 increased levels in cecal contents after recovery. Persistence of local cytokine production in shigellosis in acute and covalescent stages was also observed in humans.^38^

Our findings suggest microbiota disruption and zinc deficiency play major roles in *S. flexneri* infection dynamics, contributing to increased *S. flexneri* colonization. Combined effects of zinc deficiency and antibiotics led to persistent colonization up to 50 d post-infection in this model. A recent study has shown that the initial status of the gut microbiome is a key factor driving host response to antibiotic treatment.^40^ Reed and colleagues showed that chronic zinc deficiency in chickens alters gut microbiota, decreasing diversity and establishing microbial profile similar to other pathological states.^41^ We also have recently shown a critical role of zinc on outcomes of other enteric bacterial infections, such as enterotoxigenic *E. coli* and *Campylobacter jejuni*.^25,26^ Further studies to elucidate the mechanisms underlying the interactions between microbiota dysbiosis and zinc deficiency and its consequences for *S. flexneri* metabolism and virulence gene expression are needed.

The importance of the micronutrient zinc for infectious diarrhea is undoubted, as the benefits of zinc treatment and the consequences of zinc deficiency for enteric infectious in humans have been described extensively.^42^ In addition, a link between environmental enteropathy and zinc deficiency has recently been described. However, little is known about the mechanisms involved.^43,44^ In this model, chronic infection outcomes, characterized by the persistent colonization and cytokine production findings, were observed in zinc-deficient mice exposed to *S. flexneri* infection. In the house chow-fed mice, there was an early biochemical response to infection including changes in energy-related pathways such as fatty acid β-oxidation, the TCA cycle, and nicotinamide metabolism. This may reflect attempts by the host to generate the necessary energy required for an appropriate immune response. While the results suggest an elevation in fatty acid β-oxidation in the house chow-fed mice 2 d pi they also indicate a decrease in fatty acid oxidation in the zinc-deficient mice 6 d pi. No metabolic differences were observed between infected and uninfected zinc-deficient mice 2 d pi confirming that fatty acid β-oxidation is not part of an early adaptive response in zinc-deficient mice. Previous studies have shown a close link between zinc status and fatty acid metabolism.^45,46^ These observations may suggest a lack of metabolic flexibility in the zinc-deficient mice hindering their ability to mount a rapid and competent immune response – similar observations were seen in a mouse model of *C. jejuni* infection.^26^ In addition, the occurrence of systemic shigellosis in zinc-deficient mice, but not in house chow-fed mice, in this model corroborates the idea of impaired host response and potential disease progression in zinc-deficient mice. Interestingly, supplementing these mice with zinc reduced *S. flexneri* stool shedding and intestinal inflammation, and improved bodyweight growth. potentially through restoring metabolic homeostasis. In children, zinc supplementation shortened duration of acute shigellosis, promoted better weight gain during recovery and improved seroconversion.^47,48^ While we acknowledge that more studies are needed to elucidate the mechanisms of zinc effects in our model, these findings provide key evidence of the effects of zinc deficiency and zinc treatment on shigellosis and environmental enteropathy. Further studies for characterizing the long-term effects of *S. flexneri* infection in zinc deficient mice are currently underway.

It is important to highlight that our model reflects the biological variability that is also seen in humans. The analyses of correlation between weight change, histopathological scores and cytokine markers (TNF-α, IL-1β and IL-10) showed that mice do not respond to infection in the same way (even within the same cage), and a slightly variable timing of the peak of infection among mice in either the same or different diet fed groups is also seen. Further research is needed to better understand the key host factors that lead to greater susceptibility to disease outcomes.

In conclusion, antibiotic-treated C57Bl/6 mice provide a new model of oral *S. flexneri* infection that mimics common human clinical outcomes, with zinc deficiency promoting prolonged infection outcomes. This model is characterized by robust intestinal inflammation, epithelial damage, biochemical alterations, rare occurrence of bacteria in the lamina propria and adherence to the mucosa and epithelium in the colon, T3SS dependent pathology and intestinal cytokine production. While further studies of the mechanisms involved in microbiota disruption and associated host and pathogen responses are needed and are underway, our findings provide a valuable tool for characterizing virulence factors, host immune and metabolic responses and vaccine testing in shigellosis.

## Material and methods

### Ethics statement

This study was carried out in strict accordance with the recommendations in the Guide for the Care and Use of Laboratory Animals of the National Institutes of Health. The protocol was approved by the Committee on the Ethics of Animal Experiments of the University of Virginia (Protocol Number: 3315). All efforts were made to minimize suffering. This protocol was approved and is in accordance with the Institutional Animal Care and Use Committee policies of the University of Virginia. The University of Virginia is accredited by the Association for the Assessment and Accreditation of Laboratory Animal Care, International (AAALAC). As in our previous published mouse models of enteric infection,^22,24,25^ we used male C57BL/6 mice (which provide a consistent model for further manipulations and inter-laboratory comparisons), 4 weeks old and ordered from Jackson Laboratories (Bar Harbor, ME). Mice were co-housed in groups of four animals per cage. The vivarium was kept at a temperature of between 68–74°F with a 14 h light and 10 h dark cycle.

### Experimental design

Mice were acclimated, fed regular diet for 2–5 d, and fed either standard rodent “House Chow“ (HC), a protein source diet without zinc (dZD), or protein (2%) deficient (dPD) diet (Research Diets, Inc.) for 2 weeks prior to infection. All diets were isocaloric and calories from fat, protein, and carbohydrates are as previously reported.^15^ Four days before infection, a broad-spectrum antibiotic cocktail (metronidazole 215 mg/L, colistin 850 U/mL, gentamicin 35 mg/L and vancomycin 45 mg/L) was given in the drinking water for 3 d, as previously published.^23,49^ The antibiotic water was removed one day prior to infection with *S. flexneri* strain 2457T (1 0^8^ CFU/mouse) by gavage. Mice were followed daily for measures of weights and stool collection. For experiments in which zinc supplementation was employed, zinc sulfate was dissolved in water and filtered before giving to mice drinking water at 150 mg/L. This concentration was based on the estimated dose/weight equivalence of the US recommended daily allowance for zinc ion.^50^

### Bacterial strains and growth

*Shigella flexneri* serotype 2a strain 2457T was used, which is widely employed for genetic studies and clinical challenge studies.^51^ A nonfunctional *mxiG* strain (*ΔmxiG*) was generated in the 2457T strain as previously published to test the influence of the type 3 secretion system (T3SS).^52^ One day before infection, overnight cultures were grown from glycerol stocks in Luria Bertani broth at 37°C. On the following day, 200 μL of the culture was added to 20 mL DMEM at 37°C in a shaking incubator for 4–5 h. OD_600_ was used for monitoring. Bacterial growth was centrifuged and resuspended in 2 mL of fresh DMEM. Plate counting was used for confirming the inoculum dose. Each infected mouse received an inoculum ~1x10^8^ *S. flexneri* in 100 µL of freshly prepared DMEM; controls received 100 µL of DMEM alone.

### Intestinal inflammation evaluation

We have previously demonstrated the strong correlation of MPO, LCN-2 and calprotectin biomarkers in human stool.^53^ In the current study, murine stools and cecal contents were used to quantify intestinal inflammation biomarkers myeloperoxidase (MPO) and lipocalin-2 (LCN) using ELISA assays. After rapid dissection of the mouse intestines, cecal contents and stool were flash frozen in LN2. At time of assay, samples were lysed in RIPA buffer (20 mM Tris, pH 7.5, 150 mM NaCl, 1% Nonidet P-40, 0.5% sodium deoxycholate, 1 mM EDTA, 0.1% SDS) containing protease inhibitors cocktail (Roche) and phosphatase inhibitors (1 mM sodium orthovanadate, 5 mM sodium fluoride, 1 mM microcystin LR, and 5 mM beta-glycerophosphate). Tissue lysates were cleared by centrifugation, and the supernatant was used for total protein measurement, cytokine measurement by Luminex assay (BioRad kits), and specific ELISAs for MPO and LCN as previously described.^15^

### *S. flexneri* stool shedding and tissue burden analysis

Bacterial DNA was extracted from stools and tissues for *S. flexneri* detection by qPCR (*ipaH* gene). DNA was isolated from fecal pellets using the QIAamp DNA stool mini kit as previously described.^54^ DNA from tissue samples was extracted from frozen tissue samples using the QIAamp DNA Tissue Kit (Qiagen). For enhancing the pathogen’s DNA extraction, we made an improvement in the original protocol: a vigorous homogenization of the samples with 300 mg of 1.0 mm zirconia beads (BioSpec) using a MiniBeadBeater (BioSpec). After extraction, DNA was eluted in 200ul Elution Buffer and stored at −20°C. Quantification of the infection was performed in a Bio-Rad CFX PCR Detection System by interpolating Ct values of each run with a standard curve of known amounts of *S. flexneri* DNA and transformed into number of organisms per milligram of sample. The master mix solution and primers concentrations were used as described elsewhere.^23^ Amplification consisted of 3 min at 95°C, followed by 40 cycles of 15 s at 95°C, 60 s at 58°C. The primer sequences used were: ipaH R 5’-GTGCAGTTGTGAGCCGTTTT-3’; ipaH F 5’-ATGCGTGAGACTGAACAGCA-3’.

### Immunostaining for *S. flexneri* localization

Paraffin sections were re-hydrated in xylene, 100%, 95% 70% ethanol and water. Antigen retrieval was conducted in antigen unmaking solution (Vector Labs, H-3300) at 95°C for 20 min. Slides were blocked in PBS 2% normal goat serum for 1 h and then incubated in PBS 2% normal goat serum with primary antibodies (1:100) against *Shigella spp*. (ViroStat, # 0901) and E-cadherin (BD Transduction Laboratories, #610181) overnight at 4°C. Slides were wash briefly in PBS and incubated for 2 h in PBS 2% normal goat serum with secondary antibodies (1:100) (Life Technologies, #P10994 and #A31555). Slides were mounted with ProLong Gold antifade reagent (Invitrogen, #P36934) and imaged on a TE2000 fluorescence microscope (Nikon).

### Histological scoring

Colon segments approximately 3 cm in length were opened longitudinally, rolled inversely onto a toothpick in a swiss roll style, fixed in 4% paraformaldehyde, embedded in paraffin, and stained with hematoxylin-eosin at the University of Virginia Histology Core. Histopathological scoring was performed by blinded investigator and based in previous studies.^55,56^ The categories: loss of mucosal architecture, mucosa thickening, mucosa cell infiltration, submucosa cell infiltration, vascular density (hemorrhage) and muscular cell infiltration were graded from 0 to 3 reflecting absent, mild, moderate or severe effects.

### ^1^H NMR spectroscopy-based metabolic profiling

Urine samples were analyzed by ^1^H nuclear magnetic resonance (NMR) spectroscopy. Each sample was prepared by combining 30 μL of urine with 30 μL of phosphate buffer (pH 7.4, 100% D_2_O) containing 1mM of the internal standard, 3-trimethylsilyl-1-[2,2,3,3-^2^H4] propionate (TSP). Samples were vortexed and centrifuged (10,000 *g*) for 10 min at 4°C before transfer to a 1.7 mm NMR tube. Spectroscopic analysis was performed at 300 K on a 600 MHz Bruker NMR spectrometer equipped with a BBI probe. Standard one-dimensional spectra of the urine samples were acquired with saturation of the water resonance, using a standard pulse sequence. For each sample, four dummy scans were followed by 64 scans collected in 64 K time domain points and with a spectral window set to 20 ppm. A relaxation delay of 4 s, a mixing time of 10 ms, an acquisition time of 2.73 s and 0.3 Hz line broadening was used. Spectra were referenced to the TSP resonance at δ 0.0. ^1^H NMR spectra (δ −0.5–10) were digitized into consecutive integrated spectral regions (~20,000) of equal width (0.00055 ppm). Spectral regions corresponding to TSP (δ −0.5–0.5), water (δ 4.5–4.8) and urea (δ 5.6–6.1) were removed. The resulting spectral data were then normalized to unit area. Multivariate statistical modeling was performed using in-house scripts in MATLAB (R2016a). This included principal components analysis (PCA) using Pareto scaling and orthogonal projections to latent structures-discriminant analysis (OPLS-DA) using data mean centering. OPLS-DA models were built to facilitate data interpretation. ^1^H NMR spectroscopic profiles (metabolic information) served as the descriptor matrix and class membership (e.g. house chow uninfected mice vs house chow *S. flexneri* infected mice) was used as the response variable. The predictive performance (Q^2^Y) of the models was calculated with the use of a sevenfold cross-validation method and model validity was evaluated through permutation testing (1000 permutations). Significant metabolites were identified and their correlation with the predictive component was extracted from valid pair-wise OPLS-DA models and summarized in heat maps.

### Statistical analysis

Data analyses were performed with GraphPad Prism 6 software (GraphPad Software). All statistical analyses were done from raw data with the use of analysis of variance, Student t-tests, and Bonferroni *post hoc* analysis where applicable. Differences were considered significant at P < 0.05. Data are represented as means ± standard errors of the mean. Data were replicated at least two times in different experiments.
