# Supplementary material for: A murine model of diarrhea, growth impairment and metabolic disturbances with Shigella flexneri infection and the role of zinc deficiency
Source: Gut Microbes. 2019 Feb 3;10(5):615–30. doi: 10.1080/19490976.2018.1564430 (PMC6748602; doi:10.1080/19490976.2018.1564430)
Supplement: Supplemental Material [file kgmi-10-05-1564430-s001.zip › 1564430_suppl. Infor/Supp. Figure legend.docx]

**Fig Supplementary 1.** Higher magnified picture of stained colon section showing *S. flexneri* in red and E-cadherin (epithelium) in green, indicating *S. flexneri* to be highly predominant as biofilm-like structures in close contact with epithelial cells in zinc deficient mice
