# Supplementary figures and images for: A murine model of diarrhea, growth impairment and metabolic disturbances with Shigella flexneri infection and the role of zinc deficiency
Source: Gut Microbes. 2019 Feb 3;10(5):615–30. doi: 10.1080/19490976.2018.1564430 (PMC6748602; doi:10.1080/19490976.2018.1564430)

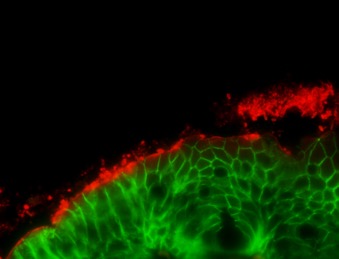

Supplement: Supplemental Material [file kgmi-10-05-1564430-s001.zip › 1564430_suppl. Infor/Fig1S.jpg]
